# mRNA Decay Proteins Are Targeted to poly(A)^+^ RNA and dsRNA-Containing Cytoplasmic Foci That Resemble P-Bodies in *Entamoeba histolytica*

**DOI:** 10.1371/journal.pone.0045966

**Published:** 2012-09-24

**Authors:** Itzel López-Rosas, Esther Orozco, Laurence A. Marchat, Guillermina García-Rivera, Nancy Guillen, Christian Weber, Eduardo Carrillo-Tapia, Olga Hernández de la Cruz, Carlos Pérez-Plasencia, César López-Camarillo

**Affiliations:** 1 Programa en Ciencias Genómicas, Universidad Autónoma de la Ciudad de México, México City, México; 2 Departamento de Infectómica y Patogénesis Molecular, Centro de Investigación y de Estudios Avanzados, Instituto Politécnico Nacional, México City, México; 3 Programa Institucional de Biomedicina Molecular y Red en Biotecnología, Escuela Nacional de Medicina y Homeopatía, Instituto Politécnico Nacional, México City, México; 4 Unité Biologie Cellulaire du Parasitisme, Institut Pasteur, Paris, France; 5 INSERM U786, Paris, France; 6 Unidad de Genómica y Secuenciación Masiva, Instituto Nacional de Cancerología, México City, México; 7 Unidad de Biomedicina, Facultad de Estudios Superiores-Iztacala, Universidad Nacional Autónoma de México, México City, México; German Cancer Research Center, Germany

## Abstract

In higher eukaryotes, mRNA degradation and RNA-based gene silencing occur in cytoplasmic foci referred to as processing bodies (P-bodies). In protozoan parasites, the presence of P-bodies and their putative role in mRNA decay have yet to be comprehensively addressed. Identification of P-bodies might provide information on how mRNA degradation machineries evolved in lower eukaryotes. Here, we used immunofluorescence and confocal microscopy assays to investigate the cellular localization of mRNA degradation proteins in the human intestinal parasite *Entamoeba histolytica* and found evidence of the existence of P-bodies. Two mRNA decay factors, namely the *Eh*XRN2 exoribonuclease and the *Eh*DCP2 decapping enzyme, were localized in cytoplasmic foci in a pattern resembling P-body organization. Given that amoebic foci appear to be smaller and less rounded than those described in higher eukaryotes, we have named them “P-body-like structures”. These foci contain additional mRNA degradation factors, including the *Eh*CAF1 deadenylase and the *Eh*AGO2-2 protein involved in RNA interference. Biochemical analysis revealed that *Eh*CAF1 co-immunoprecipitated with *Eh*XRN2 but not with *Eh*DCP2 or *Eh*AGO2-2, thus linking deadenylation to 5′-to-3′ mRNA decay. The number of *Eh*CAF1-containing foci significantly decreased after inhibition of transcription and translation with actinomycin D and cycloheximide, respectively. Furthermore, results of RNA-FISH assays showed that (i) *Eh*CAF1 colocalized with poly(A)^+^ RNA and (ii) during silencing of the *Ehpc4* gene by RNA interference, *Eh*AGO2-2 colocalized with small interfering RNAs in cytoplasmic foci. Our observation of decapping, deadenylation and RNA interference proteins within P-body-like foci suggests that these structures have been conserved after originating in the early evolution of eukaryotic lineages. To the best of our knowledge, this is the first study to report on the localization of mRNA decay proteins within P-body-like structures in *E. histolytica*. Our findings should open up opportunities for deciphering the mechanisms of mRNA degradation and RNA-based gene silencing in this deep-branching eukaryote.

## Introduction

The control of messenger RNA (mRNA) degradation plays a key role in the posttranscriptional regulation of gene expression [1]. In eukaryotic cells, mRNA degradation is initiated by shortening of the 3′ end-poly(A)^+^ tail by the CCR4/CAF1/NOT1–5 complex and the PAN2, PAN3 and PARN deadenylases [2]. Next, the DCP1 and DCP2 decapping enzymes remove the cap from the 5′ end of the deadenylated mRNA in a process stimulated by Dhh1, Edc3, Pat1/Mrt1, Sm-like (Lsm1p–7p) and Hedls proteins [3]. Lastly, the uncapped mRNA body is degraded by XRN1 and XRN2 exoribonucleases in the 5′-to-3′ direction. Alternatively, mRNA is degraded in the 3′-to-5′ direction by a complex of PH-like ribonucleases known as the exosome [4].

There is growing body of evidence to show that mRNA degradation occurs in specialized cytoplasmic foci variously referred to as mRNP granules, mRNA-decay foci, GW182-bodies and mRNA processing bodies (P-bodies). These structures are enriched in RNA substrates and mRNA turnover proteins. P-bodies are mRNA processing centers within which non-translating transcripts are sorted and either silenced or degraded [5]. Although the protein inventory of P-bodies has not been defined in detail, around 25 different factors have been detected within these small cytoplasmic domains [6]. The P-bodies observed in yeast, insect, nematode and mammalian cells have critical roles in mRNA degradation, mRNA storage, mRNA surveillance and RNA-based gene silencing mechanisms [7]. In fact, it has been demonstrated that P-bodies have high concentration of target transcripts and the AGO2, DICER, and GW182 proteins involved in RNA interference by microRNAs (miRNAs) and small interfering RNAs (siRNAs) [8], [9], [10]. In addition, P-bodies also contain the UPF and SMG families of proteins involved in the nonsense mediated decay (NMD) mechanism that degrades aberrant mRNAs containing premature translation termination codons. [11]. In mammals, most cells contain from three to nine P-bodies, although this number appears to depend on the cell's proliferation ratio and stress conditions, such as nutrient deprivation, oxidative stress and heat shock. Cell treatments with inhibitors of transcription or translation (such as actinomycin D and cycloheximide, respectively) lead to the disassembly of P-bodies [12]. In contrast, treatment with puromycin increases the number and size of P-bodies; this observation emphasizes the relevance of active mRNA and protein synthesis in the assembly of these structures [10]. Even though P-body components clearly play crucial roles in mRNA degradation, there is also evidence to show that NMD and silencing mediated by miRNAs and siRNAs occur in cells lacking detectable P-bodies. This suggests that P-body formation is a consequence, rather than a cause, of mRNA degradation activity [10], [13]. Furthermore, translational repression by miRNAs does not require P-body structures; the localization of miRNAs and RNA-induced silencing complex (RISC) proteins within P-bodies appears to be a consequence of translational repression [14].

Human amebiasis is caused by infection with the intestinal protozoan parasite *Entamoeba histolytica*. The disease is endemic in many developing countries and affects around 50 million people worldwide [15]. The symptoms of amebiasis include colitis, dysentery and extra-intestinal abscesses. Despite the importance of post-transcriptional events in the regulation of gene expression, the parasite's mRNA degradation and RNA-based gene silencing mechanisms are poorly understood. Some proteins involved in the RNA interference pathway, including *Eh*AGO2-2, *Eh*RNAseIII and *Eh*RdRP, have been identified and characterized in *E. histolytica* [16], [17]. However, proteins involved in mRNA decay pathways have not previously been studied and the potential role of P-bodies in mRNA decay and RNA interference has not been clearly established in protozoan parasites. Here, we sought to localize mRNA degradation factors within *E. histolytica* and found evidence of the existence of P-body-like structures. Our findings open up opportunities for deciphering the mechanisms of mRNA decay and RNA gene silencing in this parasitic, deep-branching eukaryote.

## Results

### mRNA degradation machineries in *Entamoeba histolytica*

**Figure 1:**
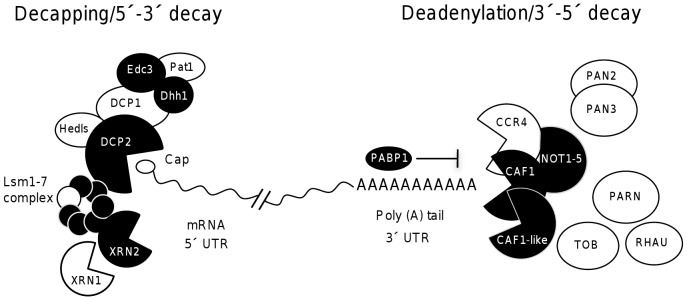
A comparative representation of mRNA decay machineries in *E. histolytica* (black shapes) and *H. sapiens* (white and black shapes). Homologous proteins were assigned via BLAST and ClustalW multiple alignments. The undulated line denotes mRNA.

In order to identify homologous genes potentially involved in mRNA decay in *E. histolytica*, we screened the parasite genome at AmoebaDB (http://amoebadb.org/amoeba/) by using the amino acid sequences of yeast and human proteins involved in mRNA turnover as probes. Heuristic searches identified several putative protein-encoding genes for mRNA degradation machineries, including deadenylation, decapping, NMD, and RNA interference activities (Figure 1, Table S1). Our data indicated that although several important genes are lacking, mRNA decay machineries are generally well conserved in *E. histolytica*. We detected putative *Ehdcp2*, *Ehlsm1–6*, *Ehedc3* and *Ehdhh1* genes (coding for mRNA decapping factors) and an *Ehxrn2* gene (coding for a putative 5′-to- 3′ exoribonuclease). In contrast, we did not find any *dcp1* or *xrn1* homologs. We also identified genes for NOT1–5 subunits and for *Eh*CAF1 and *Eh*CAF1-like deadenylases. In contrast, the lack of a *ccr4* deadenylase homolog suggested the existence of a CAF1/NOT complex lacking one of the major deadenylases. Intriguingly, we did not detect *pan2*, *pan3* and *parn* genes; this indicates that *E. histolytica*'s deadenylation machinery is smaller than those described in yeast and in the human. Amoeba genome also contains genes for the NMD machinery, as represented by three *Ehupf* genes (Table S1). RNA interference pathway members, including *Eh*RdRP, *Eh*RNAseIII and *Eh*AGO2-2 proteins, have already been reported in this parasite [16], [17]. We (like others) did not detect DICER and GW182 homologs, suggesting that RNA interference may occur by small interfering RNAs generated by DICER-independent mechanisms in *E. histolytica* [18].

### mRNA degradation genes are differentially expressed under diverse stress conditions

**Figure 2:**
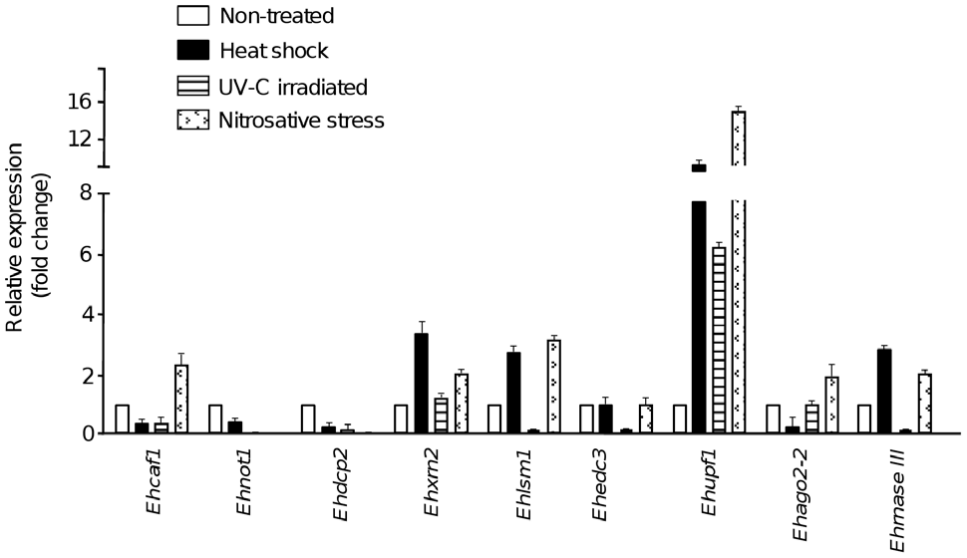
mRNA expression profiles of genes for *E. histolytica* mRNA degradation proteins. Quantitative real-time PCR assays were performed to analyze the relative expression of representative mRNA degradation genes after heat shock, UV-C induced DNA damage and sodium nitroprusside treatments. For each triplicate experiment, the mean of the relative concentrations obtained for the tested mRNA were divided by the mean of the corresponding values obtained for endogenous ribosomal *L31* amplification. Each PCR experiment was carried out three times and three independent biological samples were analyzed. The relative expression (fold change) of mRNA degradation genes in the different treatments was calculated by the 2(−ΔΔCt method) using as reference the Ct data for the untretated condition. Bars indicate the mean ± SD.

We next sought to establish whether mRNA degradation genes are transcribed in *E. histolytica* trophozoites grown in basal culture conditions and how these genes are modulated in response to the stress induced by heat shock, UV-C-induced DNA damage and nitric oxide treatments, as described elsewhere [19], [20], [21]. We designed specific primers (Table S2) and performed quantitative real time-PCR assays for representative genes from the various mRNA degradation machineries (Figure 2). After heat shock, *Ehxrn2, Ehlsm1, Ehupf1*, and *Ehrnase III* gene expression was found to be upregulated 1.5- to 12-fold relative to non-treated cells, whereas the *Ehcaf1, Ehnot1, Ehdcp2* and *Ehago2-2* genes were repressed 2-fold (Figure 2). After UV-C-induced DNA damage, *Ehupf1* was upregulated by a factor of 6, whereas the expression of *Ehcaf1, Ehnot1, Ehdcp2*, *Ehlsm1, Ehedc3* and *Ehrnase III* genes was significantly repressed. *Ehxrn2* and *Ehago2-2* mRNA levels were similar in treated and non-treated cells. *Ehnot1* and *Ehdcp2* genes were significantly repressed after exposure to nitrosative stress in comparison with untreated trophozoites, whereas *Ehcaf1, Ehlsm1, Ehupf1, Ehago2-2* and *Ehrnase III* were upregulated 2- to 16-fold. We quantified the mRNA expression of the L31 ribosomal protein-encoding gene as an endogenous control; its expression did not change significantly under any of the tested stress conditions.

### *Eh*XRN2 and *Eh*DCP2 have the typical architecture of a 5′-3′ exoribonuclease and a decapping protein, respectively

**Figure 3:**
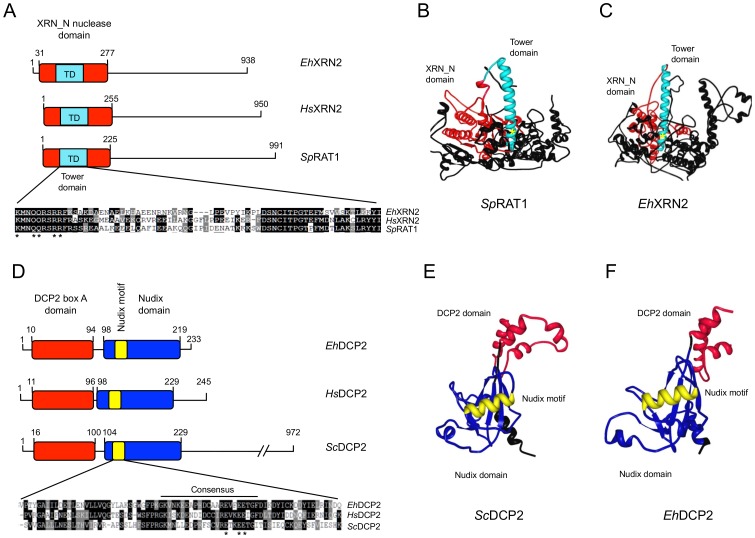
Comparison of XRN2 and DCP2 from eukaryotic organisms and *E. histolytica*. (A) A schematic representation of XRN2 protein from *E. histolytica*, *H. sapiens* and *S. pombe*. Red box: the XRN domain; cyan box: the tower domain (TD). The numbers are relative to the initial methionine in each protein. Lower panel: multiple alignments of amino acid residues from the TD of homologous proteins. Black box: a conserved residue; grey box: a conserved substitution. Asterisks mark the conserved residues in the active site. (B, C) Ribbon diagrams of *Sc*RAT1's tertiary structure (B) and the predicted model of *Eh*XRN2 (C). Red: the N-terminal XRN_N domain; cyan: the TD. (D) A schematic representation of the DCP2 protein from *E. histolytica*, *H. sapiens*, and *S. cerevisiae*. Red box: the DCP2 domain; blue box: the nudix domain. The numbers are relative to the initial methionine in each protein. Lower panel: multiple alignments of amino acid residues from the nudix motif (PF00293) of homologous proteins. Asterisks indicate the conserved residues involved in catalytic activity. Black box: a conserved residue; grey box: a conserved substitution. (E, F) Ribbon diagrams of *Sc*DCP2's tertiary structure (E) and the predicted model of *Eh*DCP2 (F). Red: the DCP2 domain; blue: the nudix domain; yellow: the nudix motif. Three-dimensional models were displayed and refined using the RCSB PBD Protein Workshop 3.9 viewer.

To initiate the study of mRNA degradation proteins in *E. histolytica*, we focused on the putative *Eh*XRN2 exoribonuclease and the *Eh*DCP2 decapping enzyme. We selected these proteins because their homologs have been described as P-body markers in the human and in yeast [22]. Homologous XRN2 and DCP2 proteins from a variety organisms were assigned by BLAST analysis and ClustalW multi-alignments of predicted amino acid sequences (Figure S1A, S2A, and Table S3, S4). *Ehxrn2* is an intronless gene (2813 nt) that predicts a 938 amino acids polypeptide. Sequence similarity searches revealed that *Eh*XRN2 exhibits 46% identity (*e* = 4.9e-139) and 45% identity (*e* = 1.2e-108) with *Homo sapiens* XRN2 and *Schizosaccharomyces pombe* RAT1, respectively. Phylogenetic inference of full-length XRN2 sequences revealed that *Eh*XRN2 is closely related to homologous proteins from other protozoa (Figure S1B). Comparison of the amino acids sequence of *Eh*XRN2 with those of *Hs*XRN2 and *Sp*RAT1 homologs indicate the presence of conserved functional and structural motifs as represented by the XRN_N nuclease domain and the internal tower domain (Figure 3A), which is responsible for catalytic activity in *Sp*RAT1 [23]. Importantly, the active site motif KX_2_QQX_2_RR, which is critical for *Sp*RAT1 ribonuclease function, is well conserved in *Eh*XRN2. The high degree of homology between *Eh*XRN2 and *Sp*RAT1 allowed us to construct a structural model of *Eh*XRN2 by using *Sp*RAT1 crystal data as template. The predicted tertiary structure suggested that the *Eh*XRN2 N-terminal domain is constituted by a six α-helix bundle and a two-stranded β-sheet, the orientation and length of which is similar to that found in the *Sp*RAT1 structural model (Figure 3B–C).

The *Ehdcp2* gene (699 nt) contains two introns and predicts an open reading frame of 233 amino acids. *Eh*DCP2 exhibits 28% (*e* = 8.4e-25) and 29% (*e* = 1.6e-19) identity with human DCP2 and yeast DCP2, respectively. Phylogenetic inference revealed that *Eh*DCP2 is closely related to homologous proteins from other protozoa (Figure S2B). *Eh*DCP2 contains the conserved DCP2 box A domain (Figure 3D) and the consensus sequence for the nudix motif GX_5_EX_7_REUXEEXGU (including the three catalytic glutamate (E) residues). In mammalian cells, both these domains are responsible for cap structure removal [24]. Modeling of *Eh*DCP2's tertiary structure using the *Sc*DCP2 structure as a template indicated that the two proteins are similar in terms of conformation and length (Figure 3E–F).

### *Eh*XRN2, *Eh*DCP2 and *Eh*CAF1 are cytoplasmic proteins

**Figure 4:**
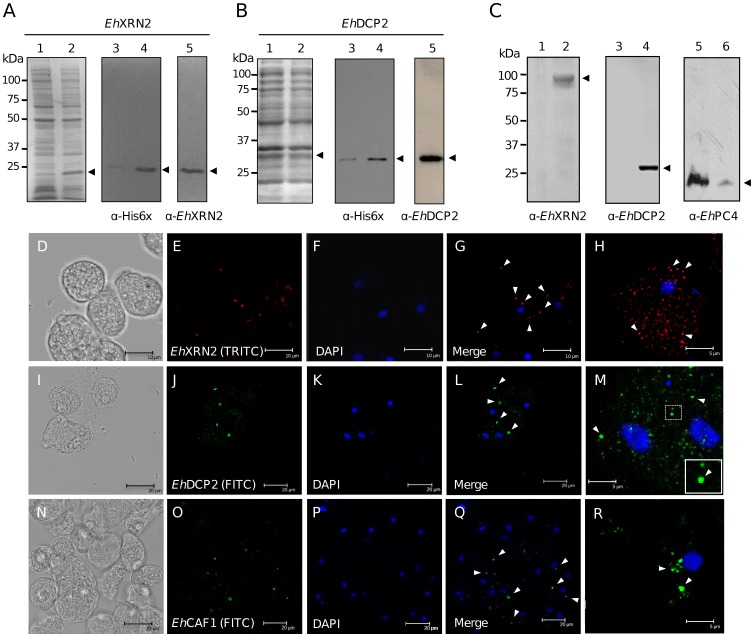
Expression and immunolocalization of *Eh*XRN2, *Eh*DCP2 and *Eh*CAF1. (A–B) Expression of 6xHis-tagged *Eh*XRN2 (A) and *Eh*DCP2 (B) proteins. Bacterial proteins were separated by 12% SDS-PAGE and the gels were stained with Coomassie blue. Lane 1: non-induced bacterial extracts; lane 2: IPTG-induced bacterial extracts. Western blot assays were performed using anti-6xHis tag antibodies and non-induced bacterial extracts (lane 3) or IPTG-induced bacterial extracts (lane 4). Recombinant *Eh*XRN2 and *Eh*DCP2 proteins were also immmunodetected with specific antibodies (lane 5). (C) Immunodetection of *Eh*XRN2 (lanes 1 and 2), *Eh*DCP2 (lanes 3 and 4) and *Eh*PC4 (lanes 5 and 6) proteins in trophozoites, using specific antibodies. Lanes 1, 3 and 5: nuclear extracts; lanes 2, 4 and 6: cytoplasmic extracts. (D–R) Confocal immunofluorescence microscopy assays for the localization of *Eh*XRN2 (E), *Eh*DCP2 (J) and *Eh*CAF1 (O) in fixed trophozoites using FITC-antibodies. Middle panels (F, K, P) show nuclear staining with DAPI and right-hand panels (G, L, Q) show overlays of the two signals. Different magnifications of single cells were used to enhance the visibility of cytoplasmic structures (H, M, R). Arrowheads indicated some typical foci.

We next investigated the cellular localization of *Eh*XRN2 and *Eh*DCP2 factors in *E. histolytica*. An *Ehxrn2* gene fragment (351 nt) and the full-length *Ehdcp2* gene were PCR-amplified from genomic DNA and cloned in-frame into the pRSET-A expression vector. Recombinant 6xHis-tagged *Eh*XRN2 and *Eh*DCP2 polypeptides were expressed in bacteria (Figure 4A and B; lane 2), purified by affinity chromatography and used as immunogens in the mouse to obtain specific antibodies. In Western blot assays based on anti-*Eh*XRN2 and *Eh*DCP2 antibodies, we detected the corresponding purified recombinant proteins (Figure 4A and B, lane 5). The subcellular localization of *Eh*XRN2 and *Eh*DCP2 were investigated by Western blotting cytoplasmic and nuclear protein extracts from *E. histolytica* trophozoites (Figure 4C). In the cytoplasmic fraction, specific antibodies recognized bands at 103 kDa and 27.5 kDa, which correspond to the predicted molecular weights for endogenous *Eh*XRN2 and *Eh*DCP2 proteins, respectively (Figure 4C, lanes 2 and 4). In contrast, no proteins in the nuclear fraction (lanes 1 and 3) were recognized. The results of control assays with used pre-immune serum (which did not give any signal; data not shown) and antibodies that recognized the *Eh*PC4 transcription factor (found mainly in the nuclear fraction (lane 5), as reported elsewhere [25]) confirmed the absence of cross-contamination between cytoplasmic and nuclear fractions.

We then used immunofluorescence and confocal microscopy assays to study the distribution of mRNA degradation proteins within trophozoites. As shown in Figure 4, *Eh*XRN2 and *Eh*DCP2 proteins were diffusely immunodetected throughout the cell (D-H and I-M) but were mainly concentrated in bright cytoplasmic foci with a diameter ranging from 50 to 300 nm; this resembled the organization into P-bodies seen in higher eukaryotes. To further investigate the localization of additional mRNA degradation proteins within trophozoites, we studied the cellular distribution of *Eh*CAF1 deadenylase using specific rabbit antibodies generated in-house (manuscript in preparation). In particular, *Eh*CAF1 had a similar pattern to *Eh*XRN2 and *Eh*DCP2 within cytoplasmic foci and a more diffuse general staining (Figure 4N–R).

### *Eh*XRN2, *Eh*DCP2 and *Eh*AGO2-2 colocalize within *Eh*CAF1-containing cytoplasmic foci

**Figure 5:**
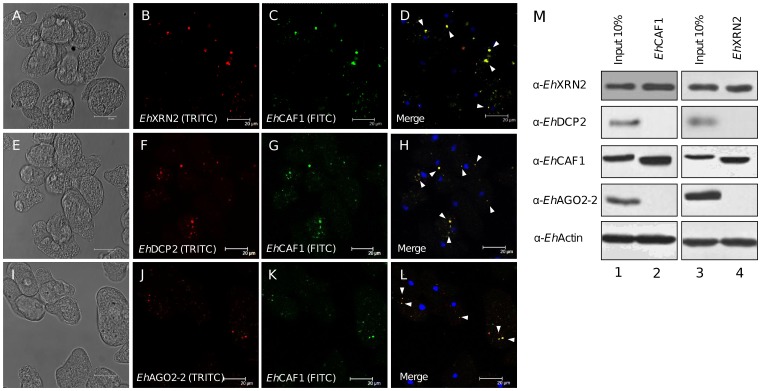
Colocalization of mRNA decay factors with *Eh*CAF1 in cytoplasmic foci. (A–L) Confocal immunofluorescence microscopy assays. Trophozoites were incubated with anti-*Eh*XRN2 (B), anti-*Eh*DCP2 (F), anti-*Eh*AGO2-2 (J) and anti-*Eh*CAF1 (C, G, K) primary antibodies (1∶100). Secondary antibodies included TRITC-conjugated anti-mouse IgG (1∶200) and FITC-conjugated anti-rabbit IgG (1∶200). (A, E and I): light microscopy; (B, F and J): the red channel; (C, G and K): the green channel; (D, H and L): merged images. Arrowheads mark typical, colocalized signals in cytoplasmic foci. (M) Immunoprecipitation assays using anti-*Eh*CAF1 and anti-*Eh*XRN2 antibodies conjugated to protein G beads with cytoplasmic lysates of *E. histolytica* trophozoites. Immunoprecipitates were separated with SDS-PAGE and analyzed by Western blotting with the antibodies indicated at the left side of each panel. Lanes 1 and 3: input (10% of the total lysate used for immunoprecipitation); lanes 2 and 4: immunoprecipitated proteins.

The observed colocalization of *Eh*XRN2, *Eh*DCP2 and *Eh*CAF1 prompted us to look for common focal, cytoplasmic structures. We performed co-immunolocalization and confocal microscopy assays in trophozoites by using *Eh*CAF1 as a marker for the P-body-like structures (Figure 5). Our data showed that both *Eh*XRN2 and *Eh*DCP2 co-immunolocalized within the majority of *Eh*CAF1-containing-cytoplasmic foci (Figure 5, A–D and E–H). We next wondered whether additional factors related to mRNA degradation might also accumulate with *Eh*XRN2 and *Eh*DCP2 in a unique set of *Eh*CAF1-containing foci. Hence, we performed immunofluorescence experiments using antibodies raised against *Eh*AGO2-2 an Argonaut protein involved in RNA interference [18]. Indeed, the *Eh*AGO2-2 signal also colocalized with *Eh*CAF1 in trophozoites (Figure 5, I–L). None of these proteins gave a significant nuclear signal. Taken as a whole, our data indicate that these mRNA degradation factors are concentrated within cytoplasmic P-body-like structures.

We next sought to establish whether the colocalized *Eh*XRN2, *Eh*DCP2, *Eh*CAF1, and *Eh*AGO2-2 proteins could interact physically by performing co-immunoprecipitation assays with anti-*Eh*CAF1 or -*Eh*XRN2 antibodies as bait and trophozoite cytoplasmic proteins as prey (Figure 5M). Our results showed that *Eh*CAF1 co-immunoprecipitated with *Eh*XRN2 and *vice versa*; these data fit with the co-immunolocalization studies and suggest a novel functional link between deadenylation and 5′-to-3′ mRNA degradation (Figure 5M, upper panel, lanes 2 and 4). In contrast, we did not detect co-immunoprecipitation of *Eh*CAF1 with either *Eh*DCP2 or *Eh*AGO2*-2* - suggesting that despite their colocalization in cytoplasmic foci in trophozoites, these proteins do not interact. Surprisingly, we also found co-immunoprecipitation of actin protein with both baits (Figure 5M, bottom panel, lanes 2 and 4). In agreement with these results, we also identified actin as an *Eh*CAF1-interacting protein in pull-down assays using GST-*Eh*CAF1 recombinant fusion protein as bait and cytoplasmic proteins as prey (manuscript in preparation). It has been reported that mammalian P-body proteins are anchored to the cytoskeleton and associate with tubulin and actin-bundles in human cells, suggesting that P-bodies require a dynamic interaction with the cytoskeleton [26].

### The formation of cytoplasmic foci depends on the presence of active transcription

**Figure 6:**
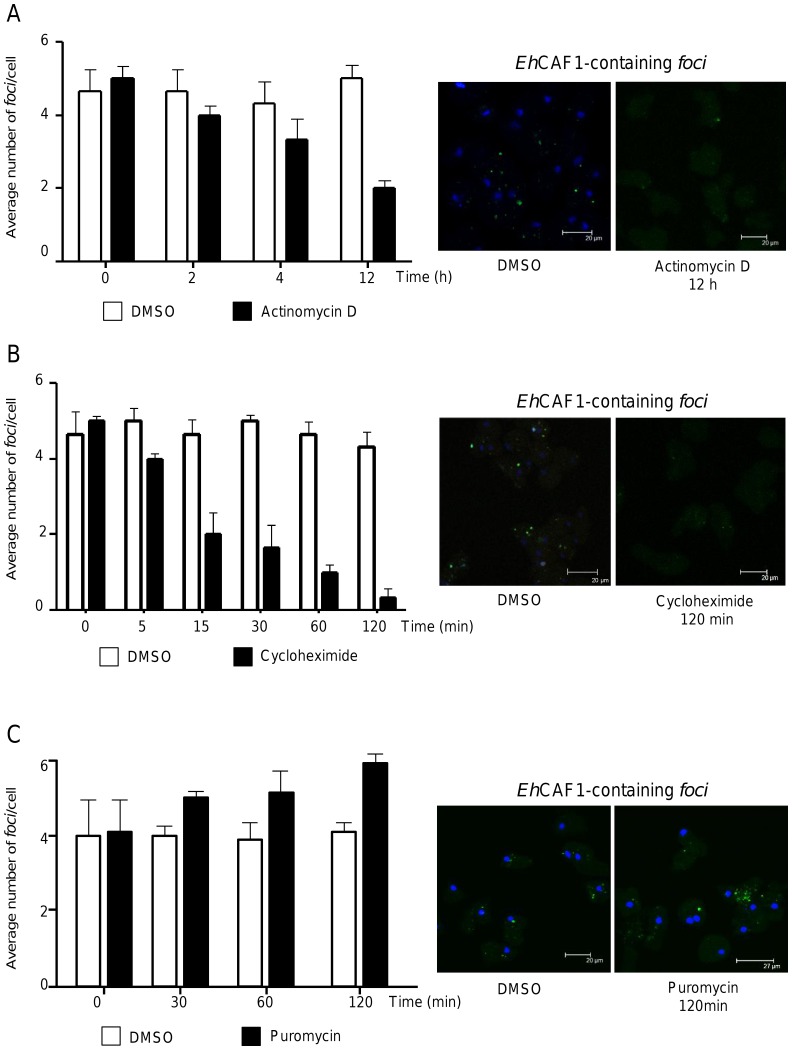
Determination of the number of *Eh*CAF1-containing cytoplasmic foci after treatment with actinomycin D, cycloheximide or puromycin. After treatment with actinomycin D (5 µg/ml, panel A), cycloheximide (10 µg/ml, panel B) or puromycin (200 µg/ml, panel C), trophozoites were fixed, incubated with anti-*Eh*CAF1 and FITC-labeled secondary antibodies, counterstained with DAPI and analyzed under an immunofluorescence confocal microscope. Left panel: change over time in the mean number of foci per trophozoite, in response to inhibition of transcription (A) or translation (B–C). The data correspond to six randomly chosen fields. Right panel: representative images of cells treated with DMSO vehicle (control) and actinomycin D, cycloheximide or puromycin (observed in the green channel).

To evaluate the impact of active ongoing transcription on P-body size and number, we measured the change over time in the number of *Eh*CAF1-containing foci after transcription inhibition with actinomycin D, as described elsewhere [27]. Our results showed that abrogation of transcription considerably altered the number of cytoplasmic foci (Figure 6A). We observed that number of cytoplasmic foci per cell diminished significantly after 4 h of incubation with actinomycin D. After 12 h treatment, the number of foci per cell had fallen by 50%. This effect was not observed in cells treated with the vehicle DMSO alone. The results of Western blot assays showed that the expression level of *Eh*CAF1 was not significantly affected by actinomycin D treatment (data not shown). Hence, the fall in the number of foci was not due to the inhibition of *Eh*CAF1 expression. Our data indicate that cytoplasmic foci formation is linked to active transcription – a feature that is conserved in P-bodies in higher eukaryotes.

### Inhibition of translation affects cytoplasmic foci formation

A common feature of mammalian P-bodies is that they are disrupted by drugs such as cycloheximide that stabilize mRNAs in polysomes, whereas their number and size are increased by drugs such as puromycin that release ribosomes from mRNA [10], [12]. To evaluate the effect of inhibiting protein synthesis on the number and size of *Eh*CAF1-containing cytoplasmic foci, we treated *E. histolytica* trophozoites with cycloheximide or puromycin. The results showed that the number of foci per cell rapidly fell after 15 min of incubation with cycloheximide and almost disappear after 120 min of treatment (Figure 6B). In contrast, puromycin treatment led to a slight increase in the number of cytoplasmic foci after 120 min (Figure 6C). Control assays showed that DMSO alone did not have a significant effect on the number of *Eh*CAF1-containing foci.

### *Eh*CAF1 and *Eh*AGO2-2 colocalize with poly(A)^+^ RNA and dsRNA substrates

**Figure 7:**
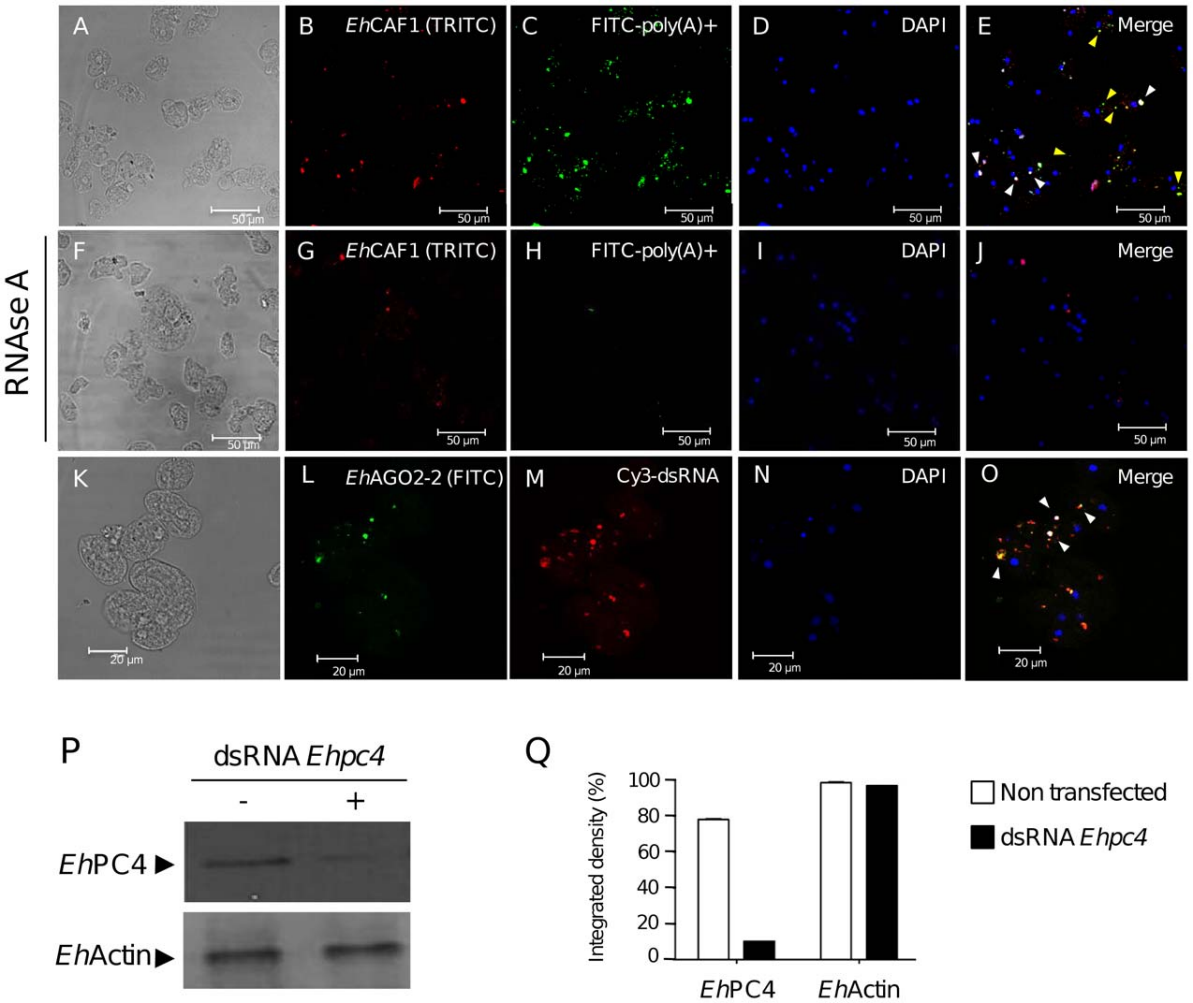
Colocalization of poly(A)^+^ RNA and dsRNA substrates with *Eh*CAF1 and *Eh*AGO2-2 in cytoplasmic foci. (A–E) Poly(A)^+^ RNA and *Eh*CAF1 colocalization assays. Trophozoites were immunostained with *Eh*CAF1 (B) antibodies. Poly(A)^+^ RNAs were detected by hybridization with FITC-conjugated oligo-(dT)_30_ (C). The cells were counterstained with DAPI (D) and analyzed with immunofluorescence confocal microscopy. The merged image (E) shows the overlapping signals. White arrowheads indicate the accumulation of poly(A)^+^ RNA and *Eh*CAF1 signals. Yellow arrowheads indicate FITC-poly(A)^+^ signal that does not overlap with *Eh*CAF1-containing foci. (F-J) RNAse A treatment, performed as a control experiment. Immunodetection of *Eh*CAF1 (G), detection of poly(A)^+^ (H) and DNA counterstaining with DAPI (I). Merged image (J). (K–O) *Eh*AGO2-2 and Cy3-dsRNA colocalization assays. Trophozoites were transfected with Cy3-dsRNA targeting *Ehpc4* (K–O) and then immunostained with *Eh*AGO2-2 antibodies on day seven after transfection (L). Cells were counterstained with DAPI (N) and analyzed with immunofluorescence confocal microscopy. The merged image (O) shows both signals. Arrowheads mark colocalized signals in cytoplasmic foci. (P) Western blot analysis for *Eh*PC4 and *Eh*Actin from proteins extracts obtained on day seven after Cy3-dsRNA transfection. (Q) A densitometric analysis of the bands in P.

We next looked at whether polyadenylated RNA substrates accumulate in P-body-like foci under basal culture conditions. Poly(A)^+^ RNAs were detected in trophozoites by fluorescence *in situ* hybridization (FISH) with a FITC-labeled oligo(dT)_30_ probe. *Eh*CAF1 foci were evidenced by immmunofluorescence with specific antibodies. Interestingly, our results showed that although the FITC-oligo(dT)_30_ signal was dispersed throughout the cytoplasm, a small proportion of probe colocalized with immunostained *Eh*CAF1 in cytoplasmic foci (Figure 7E). Control experiments showed that the FITC-oligo(dT)_30_ signal was specific for RNA, since it disappeared after RNAse A treatment (Figure 7F–J).

To gain insights into the potential role of P-body-like structures in RNA interference, we sought to determine whether a specific double-stranded RNA (dsRNA) accumulated in cytoplasmic foci and colocalized with *Eh*AGO2-2 during silencing of an endogenous gene. The long dsRNA was designed to specifically knock down the *Ehpc4* gene expression and its efficacy was validated by using the soaking approach described for amoeba [28]. Briefly, dsRNA was generated in bacteria, purified, Cy3-labeled and transfected into trophozoites to produce fluorescent siRNA. Our data showed that the dsRNA-Cy3 signal was found in discrete, cytoplasmic foci seven days after transfection of the trophozoites (Figure 7M). Moreover, coupled FISH and immunofluorescence assays using the dsRNA-Cy3 probe and *Eh*AGO2-2 antibodies showed that the two signals colocalized in large, cytoplasmic foci (Figure 7K–O). Remarkably, the focus size and signal intensity for *Eh*AGO2-2 were greater in cells than in non-transfected, control cells - suggesting that *Eh*AGO2-2 protein expression was induced by siRNA transfection. Western blot assays confirmed that *Eh*PC4 protein levels were reduced by 50% and 80% three and seven days after dsRNA-Cy3 transfection, respectively. This indicated that RNA interference had been successful (Figure 7P–Q). We can rule out the possibility that the cytoplasmic foci containing siRNA against the *Ehpc4* gene were non-transfected vesicles or endosomes, since we used the soaking method to deliver the dsRNA-Cy3 probe to trophozoites. However, we cannot be sure that only the siRNA guide strand was located in the cytoplasmic foci, since it has been reported that siRNAs can localize in P-bodies as dsRNA [29].

## Discussion

mRNA decay pathways and machineries have been extensively studied in *S. cerevisiae* and *H. sapiens*. In contrast, little is known about mRNA degradation factors in protozoan parasites. Here, we identified protein-encoding genes for mRNA degradation machineries in *E. histolytica*. Our identification of decapping, deadenylation, NMD and RNA interference activities suggests that mRNA degradation components and mechanisms are conserved in this parasite. However, the composition of these machineries is simpler than that seen in higher eukaryotic cells - a frequent feature in the small genomes of divergent parasites. Importantly, we found that the deadenylation machinery is incomplete in *E. histolytica* because we did not find *ccr4*, *pan2*, *pan3*, or *parn* deadenylases; this result suggests that *Eh*CAF1 is the parasite's main poly(A) ribonuclease. Furthermore, the decapping machinery lacks *dcp1* and *xrn1* genes but contains DCP2 and XRN2 homologs, which are typical P-body markers. Interestingly, the *Eh*XRN2 and *Eh*DCP2 proteins involved in 5′-to-3′ mRNA decay accumulated in discrete cytoplasmic foci in a manner similar to that seen in P-bodies in higher eukaryotes. This localization pattern was also observed for *Eh*CAF1 deadenylase and *Eh*AGO2-2. Given that DCP2, XRN2, CAF1 and AGO2 are resident P-body proteins in human cells, our data suggest that the foci observed in *E. histolytica* correspond to P-bodies. However, the amebic foci are smaller and less rounded than P-bodies and so we prefer to refer to them as P-body-like structures. Given the similarity between proteins involved in mRNA decay in the human, in yeast and in *E. histolytica* and the colocalization of *Eh*CAF1 with *Eh*XRN2 and *Eh*AGO2-2 in cytoplasmic structures, our results also suggest that mRNA degradation occurs at these foci. However, this hypothesis requires formal confirmation through additional experiments designed to evidence the degradation of RNA substrates and the localization of other mRNA decay proteins in these P-body-like structures.

Cytoplasmic foci resembling P-bodies have previously been described in the protozoan parasite *Trypanosoma*. Cassola *et al.* [30] reported that mRNA and proteins related to mRNA metabolism were concentrated in cytoplasmic mRNA granules in starved *T. cruzi* and *T. brucei* parasites and suggested that the said granules provided transient protection of transcription during periods of stress. However, these structures may not represent typical P-bodies. A complex containing CAF1, NOT1, NOT2, NOT5, DHH1 and a possible homolog of Caf130 was found in *T. brucei*, in which CAF1 deadenylation activity was shown to be essential for cell survival [31]. In particular, CAF1 protein appeared to be predominantly distributed in the cytoplasm and the characteristic, punctate staining indicative of association with RNA storage or degradation granules was not found. Holetz *et al.* [32] showed that the TcDhh1 helicase protein was present in polysome-independent complexes and in discrete cytoplasmic foci resembling P-bodies. The latter varied in number as a function of the cell's nutritional status and following cycloheximide and puromycin treatments. Despite these initial efforts, the existence and potential roles of P-bodies in mRNA degradation events in protozoan parasites have not been comprehensively addressed.

We cannot rule out the existence of other RNA-containing foci in *E. histolytica*, such as the stress granules described in eukaryotic cells. Stress granules are cytoplasmic aggregates of stalled 48S pre-initiation complexes. They include small ribosomal subunits, translation initiation factors (eIF2, eIF3, eIF4, eIF4G), PABP, p54/Rck and the TTP and CPEB RNA binding proteins that regulate mRNA translation and decay [33], [34]. Although P-bodies and stress granules share some proteins (e.g. TTP, p54/Rck, Lsm1, and Xrn1), there are some notable differences; P-bodies contain components of the mRNA decay pathway, whereas stress granules contain components of the translation initiation machinery and other specific markers (including TIA1) [35], [36]. The presence of stress granules in *E. histolytica* remains to be established; studies with specific markers would be required to gain a comprehensive view of cytoplasmic structures containing RNA binding proteins in this parasite.

In mammalian cells, treatment with actinomycin D induces the loss of P bodies [12], suggesting that the latter disaggregate once mRNA transcripts are no longer available for degradation. In contrast, the fact that trypanosome mRNA granules persist after actinomycin D treatment suggests that transcript degradation does not take place in these structures [30]. P-body formation in human and yeast also depends on the presence of elevated levels of non-translating mRNAs, which indicates that mRNAs exit the translation cycle before they enter P-bodies [37], [38]. The observed effects of transcriptional and translational inhibitors that are known to inhibit mRNA decay suggest that cytoplasmic foci represent sites in which mRNA decay occur. Our data revealed a similar situation in *E. histolytica* trophozoites, since the number of P-body-like structures number fell after treatment with actinomycin D and cycloheximide. It has been reported that the translation inhibitor puromycin induces an increase in the number and size of P-bodies by releasing ribosomes from mRNA. In *E. histolytica* trophozoites, the number of P-body-like structures increase slightly after puromycin treatment. Hence, our data indicate that P-body-like structures are dynamic and are associated with ongoing mRNA synthesis and translation in *E. histolytica*.

The co-immunolocalization of the deadenylase *Eh*CAF1 and the exoribonuclease *Eh*XRN2 in cytoplasmic foci in *E. histolytica* supports the hypothesis whereby P-body-like structures have an important role in mRNA decay. Furthermore, these results constitute the first evidence of a functional interaction between deadenylation and 5′-to-3′ mRNA degradation. The interaction between actin and both *Eh*CAF1 and *Eh*XRN2 further suggests that the proteins in amoebic P-body-like structures are associated with the cytoskeleton, as is the case in the human [26]. Additional, ongoing experiments should help us to understand the implications of these interactions within mRNA degradation machineries.

A small proportion of polyadenylated RNAs was concentrated in cytoplasmic foci, along with *Eh*CAF1. In addition, fluorescent siRNAs were rapidly localized with *Eh*AGO2-2 protein in cytoplasmic foci during RNA interference of *Ehpc4* gene; this observation implies that P-body-like structures may have an important role in siRNA-mediated gene silencing by facilitating the interaction between siRNAs and Argonaut proteins. It was recently reported that *Eh*AGO2-2 protein was localized in nuclei with siRNAs containing 5′-polyphosphate termini during transcriptional gene silencing in *E. histolytica* strain G3 [39]. Surprisingly, we did not detect a nuclear signal for *Eh*AGO2-2. This may be due to the fact that depending on the siRNAs generated during RNA interference, Argonaut proteins can downregulate gene expression by transporting specific classes of small regulatory RNAs to distinct cellular compartments.During transcriptional silencing, *Eh*AGO2-2 specifically transports small regulatory RNAs to nuclei in which gene silencing is mediated by histone modifications [39]. Our results indicate that both *Eh*AGO2-2 and specific siRNAs are preferentially targeted to the cytoplasm during RNA interference.

Taken as a whole, our present results provide evidence to suggest that mRNA degradation proteins involved in deadenylation and decapping pathways are organized within cytoplasmic, P-body-like structures that can be observed under the microscope. Future work is needed to understand the composition of these structures and define the localization of other mRNA decay proteins from NMD and RNA interference machineries and thus provide a better understanding of mRNA decay in this protist. This research should also provide us with an opportunity to answer intriguing, fundamental questions about the posttranscriptional regulation of gene expression in this deep-branching eukaryotic parasite.

## Materials and Methods

### Ethics statement

This study was carried out in strict accordance with the recommendations of the Guide for the Use of Laboratory Animals of the Investigation Center and Advanced Studies of the National Polytechnic Institute. The protocols and experiments were approved by the Institutional Animal Care of the Investigation Center and Advanced Studies of the National Polytechnic Institute. Animals were kept in environmentally controlled animal facilities at the Investigation Center and Advanced Studies of the National Polytechnic Institute. All surgery was performed under sodium pentobarbital anesthesia and efforts were always made to minimize suffering.

### Bacterial strains

*Escherichia coli* strain BL21pLysS was used for generation of *Eh*DCP2, *Eh*XRN2 and *Eh*AGO2-2 recombinant proteins. We used the RNAse-III deficient *E. coli* strain HT115 (rnc14::VTn10) to generate long dsRNAs targeting the *Ehpc4* gene [40].

### *E. histolytica* cultures

Trophozoites of HM1-IMSS strain were axenically cultured in TYI-S-33 medium [41]. Cells in the logarithmic growth phase were used in all experiments. Cell viability was monitored by microscopy using the trypan blue dye exclusion method.

### *In silico* identification of mRNA degradation genes

Identification of mRNA decay machinery genes was performed by screening the *E. histolytica* genome sequence at AmoebaDB (http://amoebadb.org/amoeba/). Human mRNA degradation protein sequences were used as queries. The amino acids sequences of orthologous *E. histolytica* proteins were aligned with those of mRNA degradation proteins from a range of organisms in the UniProt Knowledgebase, using ClustalW software with gap penalties of 10. Structural domains and sequence patterns were predicted with Scan Prosite.Three-dimensional structures for *Eh*XRN2 and *Eh*DCP2 were predicted with the SWISS-MODEL program by using the *S. pombe* RAT1 crystal structure (PDB 3FQD) and the *S. cerevisiae* DCP2 crystal structure (PDB 2KQMB) as a template, respectively. Three-dimensional models were displayed and refined using the RCSB Protein Data Bank Protein Workshop 3.9 viewer.

### Quantitative real-time PCR assays

Total RNA was obtained using Trizol (Invitrogen) from HM1-IMSS strain trophozoites grown in basal culture conditions and after the induction of cell stress by heat shock, DNA damage and nitric oxide treatment. cDNAs were synthesized using 1 µg total RNA, 100 ng oligo (dT_18_), 100 mM DTT, 10 mM dNTPs, 40 U RNAse inhibitor (Promega) and 200 U of Superscript II reverse transcriptase (Invitrogen) in first-strand buffer at 42°C for 90 min. After primer-specific reverse transcription, quantitative real-time PCR was performed with the primers listed in Table S2. Three independent biological samples were analyzed.

Quantitative real-time RT-PCR was carried out using an ABI PRISM 7000 Sequence Detection System (Applied BioSystems). Reactions contained 500 nM of each primer, 1× PCR SYBRGreen Master Mix (Qiagen) and 1 µL of template cDNA. Primers used for amplification are listed in Table S2. After an initial denaturation for 15 min at 95°C, the following amplification cycle (repeated 40 times) was applied: 15 s at 95°C, 15 s at 52°C and 15 s at 72°C. Next, a denaturation curve was established (55°C to 95°C, 2°C min^−1^) to check that a unique amplicon was produced. For each triplicate, the mean of the relative concentrations obtained for the tested mRNA was divided by the mean of the corresponding values obtained for amplification of the endogenous normalizer ribosomal *L31* gene. This ratio represents the variation of the mRNA abundance in the sample condition, compared with control. Each PCR experiment was repeated three times and three independent biological samples were analyzed. The relative expression of the genes coding for mRNA degradation enzymes was calculated using the 2(−ΔΔCt method) [42].

### Expression and purification of recombinant proteins

A 351-base-pair internal region of the *EhXrn2* gene (EHI_133330) was PCR-amplified from HM1-IMSS genomic DNA using *Ehxrn2-S* (5′-CCGGATCCAGGGAACAACCTCCTGTA-3′) and *Ehxrn2-AS* (5′-CCAAGCTTTAACAGTAGTTGAAAAAACCAC-3′) primers. The full-length 699 nt *Ehdcp2* gene (EHI_0 58810) was PCR-amplified from cDNA using *Ehcdp2-S* (5′-CCCGGATCCATGGATACTCATGTTCCA-3′) and *Ehdcp2-AS* (5′-CCAAGCTTTCATCTGGACTTCTTTATT-3′) primers. *Bam*HI and *Hind*III restriction sites are underlined. Amplified PCR products were purified and cloned in-frame into the pRSETA expression vector (Invitrogen). Plasmid constructions were automatically sequenced in an ABI-PRISM 310 sequencer (Applied Biosystems). The *Ehago2-2* gene was subcloned into pRSET-A from the pKT-3M plasmid (generously provided by Dr. Upinder Singh, Stanford University, CA). Recombinant *Eh*XRN2, *Eh*DCP2 and *Eh*AGO2-2 proteins were expressed as His6x-fusion polypeptides in *E. coli* strain BL21 (DE3) pLysS using 1 mM isopropyl-beta-D-thiogalactopyranoside (IPTG) for 3 hours at 37°C. Cells were disrupted by sonication and soluble *Eh*XRN2, *Eh*DCP2 and *Eh*AGO2-2 proteins were purified to near-homogeneity under native conditions with Ni-NTA sepharose chromatography (Qiagen). The identity and integrity of recombinant proteins were confirmed by Western blot assays using anti-His6x antibodies (1∶10,000) and the ECL-Plus detection system (GE Healthcare).

### Generation of antibodies and Western blot assays

After separation by SDS-PAGE, recombinant *Eh*XRN2, *Eh*DCP2 and *Eh*AGO2-2 proteins were electro-eluted from the Coomassie Blue-stained gel and used to immunize pathogen-free BALB/c male mice (*Eh*XRN2, *Eh*DCP2) or a rabbit (*Eh*AGO2-2) as follows. An initial dose of 20 µg (mice) or 150 µg (rabbit) of recombinant protein in complete Freund's adjuvant (Gibco) was injected subcutaneously inoculated into the animals. Next, three doses of 20 µg (mice) or 100 µg (rabbit) in incomplete Freund's adjuvant (Gibco) were injected via the same route at 21-day intervals. One week after the last immunization, mice were bled and antiserum was obtained. The specificity of each serum was confirmed by Western blot assays using recombinant polypeptides separated on 10% SDS-PAGE (30 µg/lane) and electrotransferred to nitrocellulose membranes. The membranes were then incubated with each immune serum (1∶1000) in 2% non-fat dried milk, 0.05% Tween-20 in PBS pH 7.4 overnight at 4°C. Proteins were developed using peroxidase-conjugated secondary antibodies (1∶5000, Zymed) and detected with the ECL-Plus system. Western blot assays using nuclear and cytoplasmic protein extracts from HMI-IMSS trophozoites were performed as described elsewhere [43].

### Immunofluorescence confocal laser assays

Trophozoites were cultured on cover slides, fixed with 4% paraformaldehyde at 37°C for 40 min and permeabilized with 0.5% Triton-X100 in PBS 1× at 37°C for 40 min. Next, cells were blocked in 2% BSA for 60 min at 37°C, washed three times with PBS 1× and incubated with either anti-*Eh*CAF1 and anti-*Eh*AGO2-2 antibodies generated in the rabbit or anti-*Eh*XRN2, and anti-*Eh*DCP2 antibodies generated in the mouse (1∶200) at 37°C for 1 h. Cells were washed three times and incubated with fluorescein-labeled secondary antibodies (1∶200) at 37°C for 1 h. Lastly, DNA was counterstained with 4′-6-diamidino-2-phenylindole (DAPI, 5 mg/ml) for 1 min. Samples were analyzed under an inverted microscope connected to a laser confocal scanning system (TCS SP2, Leica Microsystems).

### Immunoprecipitation assays

The immunoprecipitation protocol was essentially that described in [44] with minimal modifications. Briefly, cytoplasmic proteins (50 µg) were diluted in 300 µl HNTG buffer (20 mM HEPES pH 7.5, 150 mM NaCl, 0.1% Triton, 10% glycerol) and incubated with 10 µl of protein G beads (Sigma) for 30 min at 4°C. Non-specifically interacting proteins were excluded by centrifugation at 3000 rpm at 4°C for 5 min. The supernatant was incubated with either anti-*Eh*CAF1 antibodies (1∶100) or anti-*Eh*XRN2 antibodies (1∶100) for 2 h at 4°C. Next, protein G-Sepharose beads were added to the samples and incubated for 16 h at 4°C. Immunoprecipitated proteins were collected by centrifugation, washed three times with HNTG buffer and then separated using 12% SDS-PAGE. Proteins were transferred to nitrocellulose membranes and detected by Western blot assays with rabbit anti-*Eh*CAF1 or mouse anti-*Eh*XRN2 antibodies.

### Bacterial expression of double-stranded RNA and trophozoites transfection

Double-stranded RNA was obtained as described in [28]. Briefly, the *Ehpc4* full coding sequence (EHI_192520) was cloned into the *Sma*I and *Xho*I restriction sites of the pL4440 plasmid vector, which is flanked by T7 promoters [45]. Competent *E. coli* HT115 cells were transformed with pL4440-*Ehpc4* and spread onto LB-agar plates containing ampicillin and tetracycline. Isolated bacterial colonies were obtained after overnight culture at 37°C. Double-stranded RNA synthesis was induced by incubation with 2 mM IPTG for 4 h at 37°C. Cy3-labeling of purified dsRNA was performed with the Silencer siRNA labeling kit (Ambion), according to the manufacturer's instructions. The reaction mixture (containing 5 µg dsRNA, nuclease free-water, labeling buffer and Cy3 labeling reagent) was incubated for 1 h at 37°C in the dark. Labeled dsRNA was precipitated with ethanol, resuspended in DEPC-treated water and immediately used in colocalization assays.

### RNA fluorescence *in situ* hybridization

To detect poly(A)^+^ RNA in fluorescence *in situ* hybridization (FISH) assays, parasites were harvested and allowed to adhere to microscope slides. Cells were fixed with 4% paraformaldehyde in PBS, denatured in 70% formamide at 70°C and incubated for 10 min with 25 mM NH_4_Cl. The cells were permeabilized and blocked for 30 min in 0.5% Triton X-100/2% BSA in PBS, followed by 2 h of prehybridization at room temperature in hybridization buffer (2% BSA, 4× SSC, 5% dextran sulfate, 35% deionized formamide and 10 U RNAse inhibitor). Hybridization was performed overnight at room temperature in a humidified culture chamber in the presence of 300 ng/ml FITC-conjugated oligo-(dT)_30_ in hybridization buffer. Slides were washed once in 4× SSC plus 35% deionized formamide and once in 4× SSC and were then counterstained with DAPI (1 mg/ml). Slides were mounted with Vectashield antifade (Vector). The subcellular localization was analyzed with a Leica TCS SP2 microscope. Merged images were obtained by superimposing image files using LCS Lite software (Leica Microsystems).

## Supporting Information

Figure S1 **XRN2 related proteins.** (A) Multiple alignments of related XRN2 proteins. (B) Phylogenetic relationships between *Eh*XRN2 and XRN2 family members. The rooted tree was created with a neighbor-joining algorithm in the Mega 5.1 program, on the basis of ClustalW alignments of full amino acid sequences. Each protein's accession number is given in brackets.
(PDF)

Figure S2 **DCP2 related proteins.** (A) Multiple alignments of related DCP2 proteins. (B) Phylogenetic relationships between *Eh*DCP2 and DCP2 family members. The rooted tree was created with a neighbor-joining algorithm in the Mega 5.1 program, on the basis of ClustalW alignments of full amino acid sequences. Each protein's accession number is given in brackets.
(PDF)

Table S1 **Predicted mRNA degradation proteins in** ***Entamoeba histolytica*** **.**
(PDF)

Table S2 **Primers used in quantitative real-time PCR assays.**
(PDF)

Table S3 **Comparison of** ***Eh*** **XRN2 with related proteins from several organisms.**
(PDF)

Table S4 **Comparison of** ***Eh*** **DCP2 with related proteins from several organisms.**
(PDF)
