# Supplementary material for: mRNA Decay Proteins Are Targeted to poly(A)+ RNA and dsRNA-Containing Cytoplasmic Foci That Resemble P-Bodies in Entamoeba histolytica
Source: PLoS One. 2012 Sep 24;7(9):e45966. doi: 10.1371/journal.pone.0045966 (PMC3454373; doi:10.1371/journal.pone.0045966)
Supplement: Figure S2 — DCP2 related proteins. (A) Multiple alignments of related DCP2 proteins. (B) Phylogenetic relationships between EhDCP2 and DCP2 family members. The rooted tree was created with a neighbor-joining algorithm in the Mega 5.1 program, on the basis of ClustalW alignments of full amino acid sequences. Each protein's accession number is given in brackets. (PDF) [file pone.0045966.s002.pdf]

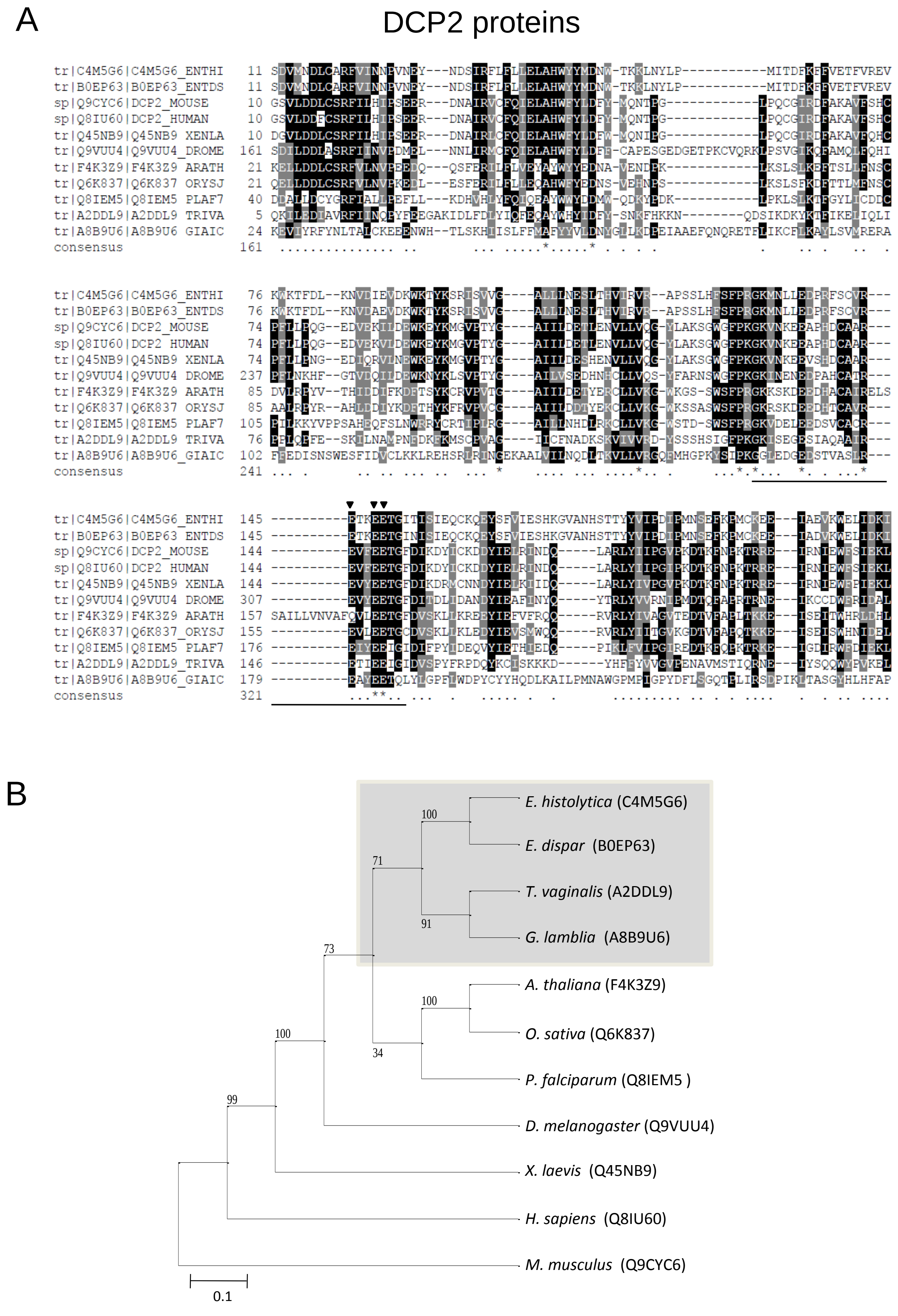
A
DCP2 proteins
tr|C4M5G6|C4M5G6_ENTHI
tr|B0EP63|B0EP63_ENTDS
sp|Q9CYC6|DCP2_MOUSE
sp|Q8IU60|DCP2_HUMAN
tr|Q45NB9|Q45NB9_XENLA
tr|Q9VUU4|Q9VUU4_DROME
tr|F4K3Z9|F4K3Z9_ARATH
tr|Q6K837|Q6K837_ORYSJ
tr|Q8IEM5|Q8IEM5_PLAF7
tr|A2DDL9|A2DDL9_TRIVA
tr|A8B9U6|A8B9U6_GIAIC
consensus
B
E. histolytica (C4M5G6)
E. dispar (B0EP63)
T. vaginalis (A2DDL9)
G. lamblia (A8B9U6)
A. thaliana (F4K3Z9)
O. sativa (Q6K837)
P. falciparum (Q8IEM5)
D. melanogaster (Q9VUU4)
X. laevis (Q45NB9)
H. sapiens (Q8IU60)
M. musculus (Q9CYC6)
100
71
91
73
100
34
100
99
0.1
