# Supplementary material for: mRNA Decay Proteins Are Targeted to poly(A)+ RNA and dsRNA-Containing Cytoplasmic Foci That Resemble P-Bodies in Entamoeba histolytica
Source: PLoS One. 2012 Sep 24;7(9):e45966. doi: 10.1371/journal.pone.0045966 (PMC3454373; doi:10.1371/journal.pone.0045966)
Supplement: Table S1 — Predicted mRNA degradation proteins in Entamoeba histolytica . (PDF) [file pone.0045966.s003.pdf]

Table S1. mRNA degradation proteins predicted in *Entamoeba histolytica*

Entamoeba histolytica						*Homo sapiens*			
Predicted protein	Size (aa)	Accession number[a]	*e*-value	I (%)	S (%)	Protein	Accession number[b]	Size (aa)	Function
Proteins involved in decapping									
-	-	-	-	-	-	DCP1	Q9NPI6	582	Decapping enzyme subunit
EhDCP2	233	EHI_058810	8.4e-25	28	50	DCP2	Q8IU60	420	Catalytic subunit of decapping enzyme
-	-	-	-	-	-	Hedls/Ge-1	Q6P2E9	1401	Component of mammalian decapping complex
-	-	-	-	-	-	RAP55	Q8ND56	463	Protein containing Sm-like and FDF domain; involved in translation repression
-	-	-	-	-	-	XRN1	Q8IZH2	1706	5’ to 3’ exoribonuclease
EhXRN2	938	EHI_133330	4.9e-139	46	64	XRN2	Q9H0D6	950	5’ to 3’ exoribonuclease
EhLsm1	110	EHI_188020	8.4e-19	31	59	Lsm1	O15116	133	Sm-like proteins involved in decapping, mRNA splicing
EhEdc3	576	EHI_198940	8.9e-10	29	45	Edc3	Q96F86	508	Decapping activator
EhDhh1	392	EHI_093900	1.7e-72	38	61	Dhh1	P26196	483	RNA helicase required for translation repression. Decapping activator
Proteins involved in deadenylation									
EhCAF1	312	EHI_048150	3.6e-56	46	63	CAF1	Q9UIV1	285	Deadenylase (CCR4-NOT complex)
EhCAF1-like	303	EHI_039000	2.9e-58	46	64	CALIF	Q9UFF9	292	Deadenylase
-	-	-	-	-	-	CCR4	Q9ULM6	557	mRNA poly (A) shortening
-	-	-	-	-	-	PAN2	Q504Q3	1202	Deadenylase
-	-	-	-	-	-	PAN3	Q58A45	887	Stimulates PAN2 nuclease activity
-	-	-	-	-	-	PARN	O95453	639	3’- exoribonuclease
EhPABP1	525	EHI_198750	5.6e-33	47	27	PABP1	P11940	636	mRNA metabolism and pre-mRNA splicing

Proteins Involved in NMD									
EhUpf1	938	EHI_035550	3.5e-36	48	62	UPF1	Q92900	1129	RNA helicase required for NMD
-	-	-	-	-	-	SMG5	Q9UPR3	1016	Factor required for dephosphorylation of UPF1
-	-	-	-	-	-	SMG7	Q92540	1137	Factor required for dephosphorylation of UPF1
RNA interference factors									
EhAGO2-2	938	EHI_125650	1.9e-23	24	42	AGO2	Q9UKV8	859	Transcriptional repressor
EhRNAse III	257	EHI_068740	7.6e-8	22	51	DICER	Q9UPY3	1922	miRNA/siRNA silencing
EhRdRP	1026	EHI_139420	-	-	-	-	-	-	-
EhRdRP	819	EHI_179800	-	-	-	-	-	-	-
-	-	-	-	-	-	GW118	Q8NDV7	1962	miRNA/siRNA silencing
-	-	-	-	-	-	TNR6B	Q9UPQ9	1833	Paralog of GW182, miRNA/siRNA silencing
-	-	-	-	-	-	MOV10	Q9HCE1	1003	Dead box helicase, miRNA/siRNA silencing
RNA- binding proteins and translation repressors									
-	-	-		-	-	CPEB	Q9BZB8	-566	Cytoplasmic polyadenylation elementbinding protein; translational regulator
EhTTP	213	EHI_008770	9.6e-24	64	71	TTP	P26651	326	AU-rich element-binding protein observed in P bodies when overexpressed
EheIF4E	201	EHI_180000	1.2e-27	35	57	eIF4E	P06730	217	Component of mammalian decapping complex
-	-	-		-	-	eIF4E-T	Q9NRA8	985	Mediates the nuclear import of eIF4E
EhGemin 5	464	EHI_130870	4.9e-12	22	38	Gemin5	Q8TEQ6	1508	eIF4E-binding protein, mRNA splicing

[a]AmoebaDB database [b]Uniprot Knowledgebase I, Identity; S, similarity NMD, Nonsense-mediated decay

For families of proteins (XRN2, Lsm 1-8, Upf1-3, eIF4E) only a representative member is shown.
