# Supplementary material for: mRNA Decay Proteins Are Targeted to poly(A)+ RNA and dsRNA-Containing Cytoplasmic Foci That Resemble P-Bodies in Entamoeba histolytica
Source: PLoS One. 2012 Sep 24;7(9):e45966. doi: 10.1371/journal.pone.0045966 (PMC3454373; doi:10.1371/journal.pone.0045966)
Supplement: Table S2 — Primers used in quantitative real-time PCR assays. (PDF) [file pone.0045966.s004.pdf]

Table S2. Primers used in quantitative real-time PCR assays

Gene	Sense primer	Antisense primer	Amplified product (bp)	Tm (°C)
Ehago2-2	5'- AAGAATCTAGCCGACGTGAC-3'	5'-TCTCTTCTTGGTGAACGGTC-3'	100	51.5
Ehdcp2	5'-TGACTTGTGTGCACGATTTG-3'	5'-GCTCCTACTACTGATATTCTTGACT-3'	267	50.8
Ehxrn2	5'-TGACAGAAGTGATAATGATATTTGC-3'	5'-TGTTTCAGGTGATTGTCGGT-3'	290	50
Ehedc3	5'- GGTTAGCTGTTGCTCAAGTTG--3'	5'-ACAAACCAACCCATCTCCTC-3'	110	51.7
Ehlsm1	5'-ACGTACATTTTCTAAAGAAGGAGTT-3'	5'-GCTTGAGAAAGTGGATTGGAAA-3'	150	51
Ehcaf1	5'-CATATCCAGGTATGGAATACCCAT-3'	5'-ATGGTGGAAATTGTCTATTGGG-3'	120	51
Ehnot1	5'-TGCTCTGAACATTTTCAAATGG-3'	5'-GACCATATCATCCATCAACGC-3'	195	50.5
Ehupf1	5'-TGAGGAAAATGGCATGGGAG-3'	5'-TGTCTATCAGCTTCAGCCTC-3'	163	51.5
Ehrnase III	5'-AGCTCAACTACATTACACAATGC-3'	5'-TGCTCGTTCTTCAAGATAGGA-3'	183	51
EhL31	5'-AGAGAGCTCCAAAAGCCATC-3'	5'-CTACGGTTTTAGTTACGAGACC-3'	244	52
