# Supplementary material for: mRNA Decay Proteins Are Targeted to poly(A)+ RNA and dsRNA-Containing Cytoplasmic Foci That Resemble P-Bodies in Entamoeba histolytica
Source: PLoS One. 2012 Sep 24;7(9):e45966. doi: 10.1371/journal.pone.0045966 (PMC3454373; doi:10.1371/journal.pone.0045966)
Supplement: Table S3 — Comparison of Eh XRN2 with related proteins from several organisms. (PDF) [file pone.0045966.s005.pdf]

Table S3. Comparison of *Eh*XRN2 with related proteins from several organisms.

Protein	Organism	Accession number[a]	E-value	I (%)	S (%)
5'-3' exoribonuclease	*Entamoeba dispar*	B0E6C7	3.4e-138	44	62
XRN 5'-3' exonuclease	*Trichomonas vaginalis*	A2DLG3	7.2e-104	52	70
Putative exoribonuclease 2	*Leishmania major*	Q4Q1P5	5.6e-60	27	46
5'-3' exoribonuclease-2	*Giardia intestinalis*	E1F363	1.6e-96	44	59
Exoribonuclease, putative	*Plasmodium falciparum*	Q8I358	7.0e-101	49	68
XRN2, 5'-3' exoribonuclease 2	*Mus musculus*	Q9DBR1	1.5e-139	46	65
XRN2, 5'-3' exoribonuclease 2	*Homo sapiens*	Q9H0D6	4.9e-139	46	64
LOC100049102 protein	*Xenopus laevis*	A3KNB2	4.9e-100	47	66
XRN2, 5'-3' exoribonuclease 2	*Drosophila melanogaster*	Q9VM71	8.2e-98	33	53
XRN2, 5'-3' exoribonuclease 2	*Schizosaccharomyces pombe*	P40848	2.1e-138	47	63
XRN2,5'-3' exoribonuclease 2	*Arabidopsis thaliana*	Q9FQ02	4.5e-134	45	62
Putative 5-3 exoribonuclease	*Oryza sativa*	Q8RV84	3.4e-138	44	62

[a]UniProtKB database. I, identity. S, similarity
