# Supplementary material for: mRNA Decay Proteins Are Targeted to poly(A)+ RNA and dsRNA-Containing Cytoplasmic Foci That Resemble P-Bodies in Entamoeba histolytica
Source: PLoS One. 2012 Sep 24;7(9):e45966. doi: 10.1371/journal.pone.0045966 (PMC3454373; doi:10.1371/journal.pone.0045966)
Supplement: Table S4 — Comparison of Eh DCP2 with related proteins from several organisms. (PDF) [file pone.0045966.s006.pdf]

Table S4. Comparison of putative *Eh*DCP2 with related proteins from several organisms.

Protein	Organism	Accession number[a]	E-value	I (%)	H (%)
mRNA-decapping enzyme	*Entamoeba dispar*	B0EP63	2.3e-122	98	99
Hydrolase, NUDIX family protein	*Trichomonas vaginalis*	A2DDL9	3.3e-14	37	48
Putative uncharacterized protein	*Giardia intestinalis*	A8B9U6	1.3e-10	30	52
NUDIX hydrolase, putative	*Plasmodium falciparum*	Q8IEM5	4.2e-19	29	49
DCP2, mRNA-decapping enzyme 2	*Mus musculus*	Q9CYC6	5.2e-25	28	50
DCP2, mRNA-decapping enzyme 2	*Homo sapiens*	Q8IU60	8.5e-25	30	50
mRNA decapping enzyme	*Xenopus laevis*	Q45NB9	9.1e-28	29	54
Decapping protein 2	*Drosophila melanogaster*	Q9VUU4	8.8e-25	32	52
mRNA-decapping enzyme subunit 2	*Arabidopsis thaliana*	F4K3Z9	4.4e-20	27	46
Decapping protein 2-like	*Oryza sativa*	Q6K837	8.7e-23	32	51

[a]UniProtKB database. I, identity. S, similarity
