# Supplementary material for: mRNA Decay Proteins Are Targeted to poly(A)+ RNA and dsRNA-Containing Cytoplasmic Foci That Resemble P-Bodies in Entamoeba histolytica
Source: PLoS One. 2012 Sep 24;7(9):e45966. doi: 10.1371/journal.pone.0045966 (PMC3454373; doi:10.1371/journal.pone.0045966)
Supplement: Figure S1 — XRN2 related proteins. (A) Multiple alignments of related XRN2 proteins. (B) Phylogenetic relationships between EhXRN2 and XRN2 family members. The rooted tree was created with a neighbor-joining algorithm in the Mega 5.1 program, on the basis of ClustalW alignments of full amino acid sequences. Each protein's accession number is given in brackets. (PDF) [file pone.0045966.s001.pdf]

A

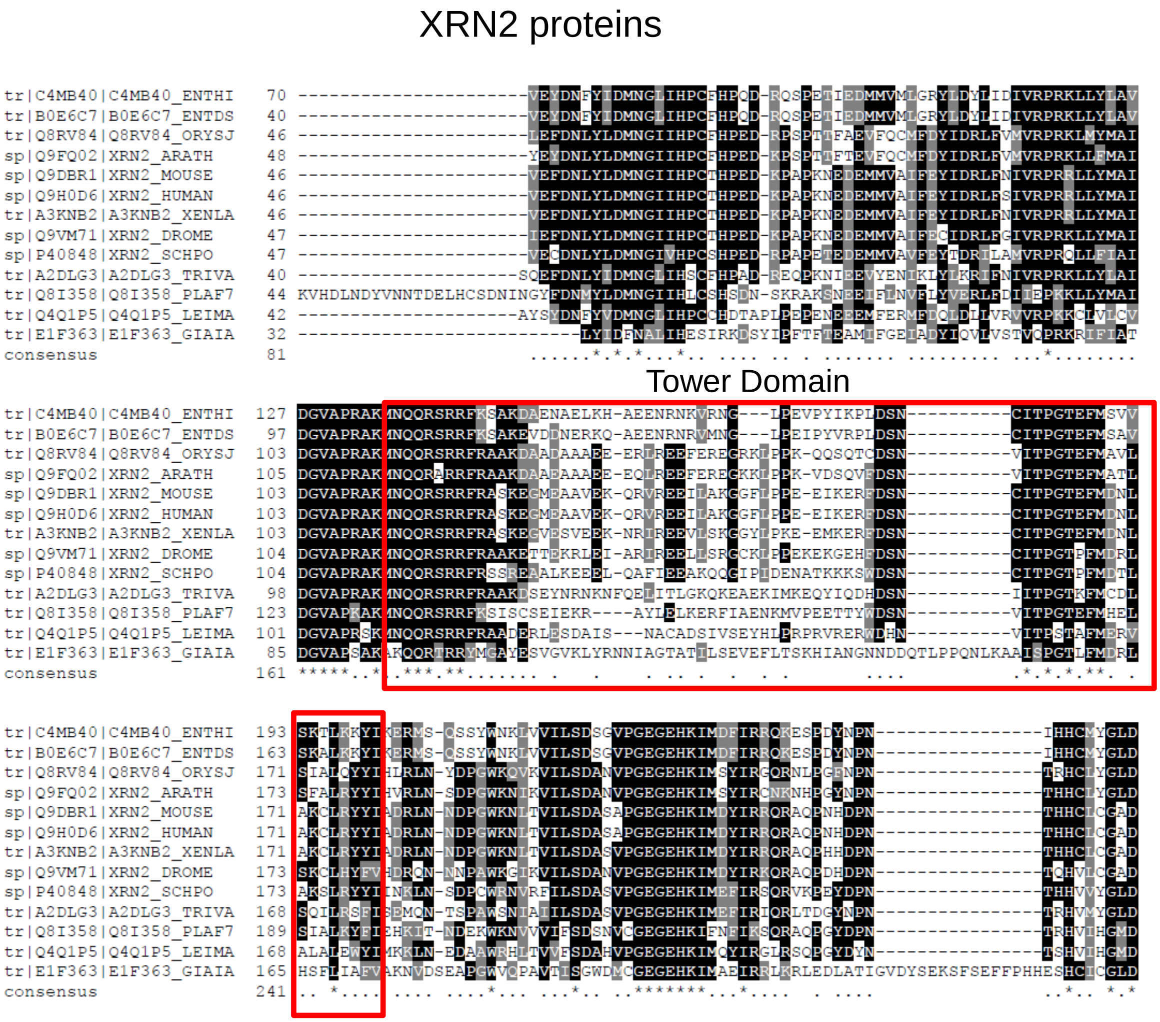
XRN2 proteins
Tower Domain
consensus

B

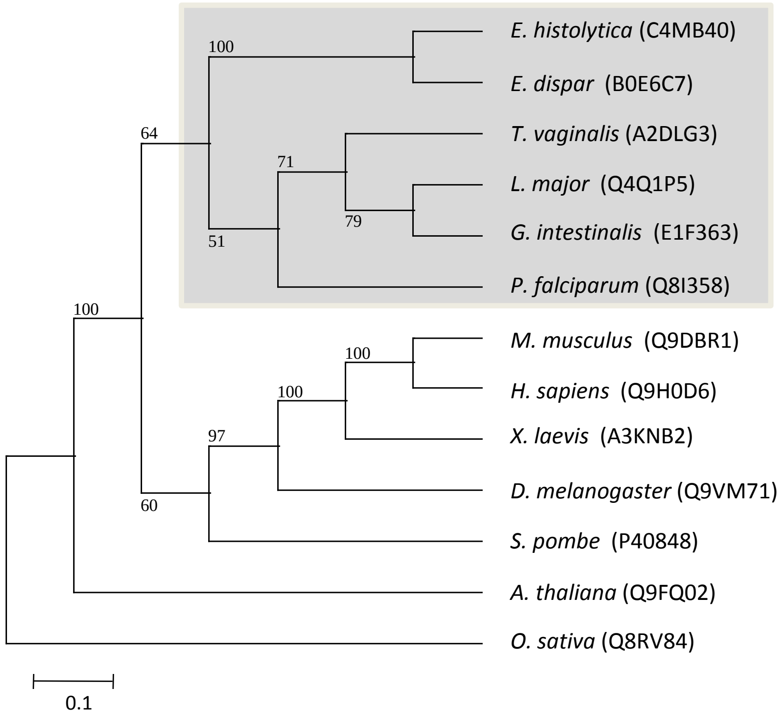
E. histolytica (C4MB40)
E. dispar (B0E6C7)
T. vaginalis (A2DLG3)
L. major (Q4Q1P5)
G. intestinalis (E1F363)
P. falciparum (Q8I358)
M. musculus (Q9DBR1)
H. sapiens (Q9H0D6)
X. laevis (A3KNB2)
D. melanogaster (Q9VM71)
S. pombe (P40848)
A. thaliana (Q9FQ02)
O. sativa (Q8RV84)
0.1

Figure S1
